# Endogenous Plasma Peptide Detection and Identification in the Rat by a Combination of Fractionation Methods and Mass Spectrometry

**Published:** 2007-10-09

**Authors:** Fabrice Bertile, Flavie Robert, Véronique Delval-Dubois, Sarah Sanglier, Christine Schaeffer, Alain Van Dorsselaer

**Affiliations:** Institut Pluridisciplinaire Hubert Curien, Département Sciences Analytiques, Laboratoire de Spectrométrie de Masse Bio-Organique, CNRS-ULP UMR 7178, ECPM, 25 rue Becquerel, 67087 Strasbourg Cedex 2, France

**Keywords:** Peptidomics, Sample fractionation, Low-abudance molecules, plasma

## Abstract

Mass spectrometry-based analyses are essential tools in the field of biomarker research. However, detection and characterization of plasma low abundance and/or low molecular weight peptides is challenged by the presence of highly abundant proteins, salts and lipids. Numerous strategies have already been tested to reduce the complexity of plasma samples. The aim of this study was to enrich the low molecular weight fraction of rat plasma. To this end, we developed and compared simple protocols based on membrane filtration, solid phase extraction, and a combination of both. As assessed by UV absorbance, an albumin depletion >99% was obtained. The multistep fractionation strategy (including reverse phase HPLC) allowed detection, in a reproducible manner (CV < 30%–35%), of more than 450 peaks below 3000 Da by MALDI-TOF/MS. A MALDI-TOF/MS-determined LOD as low as 1 fmol/μL was obtained, thus allowing nanoLC-Chip/MS/MS identification of spiked peptides representing ~10^−6^% of total proteins, by weight. Signal peptide recovery ranged between 5%–100% according to the spiked peptide considered. Tens of peptide sequence tags from endogenous plasma peptides were also obtained and high confidence identifications of low abundance fibrinopeptide A and B are reported here to show the efficiency of the protocol. It is concluded that the fractionation protocol presented would be of particular interest for future differential (high throughput) analyses of the plasma low molecular weight fraction.

## 1. Introduction

A biomarker can be defined as a detectable change in a given biological system that conveys information about biological state. Biomarkers then group together molecules whose levels can be used as indicators of a particular disease state and/or help get further insights in the molecular mechanisms potentially involved in various pathophysiological processes. Proteins and peptides are of paramount importance for most of the physiological processes. With the recent advances in mass spectrometry techniques for protein and peptide analysis, proteomic and peptidomic analyses have naturally emerged as essential tools in the field of biomarker research. In this framework, plasma or serum are being referred as the most valuable samples for biomarker discoveries. However, detection and characterization of low abundance and/or low molecular weight plasma peptides is challenged by the presence of highly abundant proteins, salts and lipids (Adkins, 2002). Therefore, reduction of the complexity of plasma samples must be considered as a step that is impossible to circumvent prior to mass spectrometry-based analyses.

At present various approaches are being used ranging from high abundance protein removal to a host of fractionation methods. High abundance protein removal is generally performed with the use of antibody-based or affinity dye-based resins (Adkins, 2002; Govorukhina, 2003; Greenough, 2004; Fountoulakis, 2004; Colantonio, 2005; Granger, 2005; Björhall, 2005; Martosella, 2005; Brand, 2006), membrane filtration (Tirumalai, 2003; Chernokalskaya, 2004; Tammen, 2005b; Tanaka, 2006; Orvisky, 2006; Ingvarsson, 2006; Zheng, 2006), and protein precipitation procedures (Merrell, 2004; Chen, 2005; Colantonio, 2005). Fractionation methods combine different separation techniques including electrophoretic (Righetti, 2005) and chromatographic (Lescuyer, 2004; Linke, 2004; Baumann, 2005). Among peptide extraction techniques, solid phase extraction (SPE) also represents an attractive approach (Souverain, 2004; Koomen, 2005). In the above mentioned studies, analyses of the plasma proteome have often combined multidimensional peptide separation strategies. Loss of proteins and peptides of interest dramatically increases as the number of sample preparation steps is increased. Therefore, cautions have to be considered regarding limited resin capacity, lack of specificity of affinity-based methods and binding of low abundance species to high abundance proteins that results in removal of potential biomarker candidates, including hormones, lipoproteins and cytokines (Schussler, 2000; Dea, 2002; Granger, 2005). Moreover, even after depletion, the remaining proteins can still be sufficiently abundant to hamper low-abundance protein biomarker determination. Despite these drawbacks, removal of abundant molecules and/or sample fractionation made MS analyses and/or identification of potential biomarkers possible in most cases (Adkins, 2002; Tirumalai, 2003; Aldred, 2004; Tammen, 2005b; Tanaka, 2006).

The aim of this study was to develop a simple protocol to avoid excess loss of material. Such a protocol was expected to allow efficient separation of low and high abundance molecules from a complex mixture, and suitable for high throughput peptidomics. Rat plasma fractionation was performed using various methodologies based on membrane filtration, SPE or a combination of both. Evaluation and comparison of these protocols were performed by determination of total protein concentration, SDS-PAGE analysis, and estimation of albumin depletion by UV absorbance. MALDI-TOF/MS analyses of fractionated samples were also compared, with determination of the number of peaks detected and of reproducibility of the experiments. Spiking of crude plasma and of fractionated samples with standard peptides were also performed to determine limit of detection (LOD) and signal recovery of spiked peptides. Finally, peptide identifications were performed with a nanoLC-Chip/MS/MS system.

## 2. Materials and Methods

### 2.1. Materials

Potassium-EDTA vacutainer^™^ tubes were purchased from Becton, Dickinson (Le Pont de Claix, France). All buffers were prepared with Milli-Q^®^ water (Millipore, Milford, MA, U.S.A.). Vivaspin^®^ 2 30,000 MWCO centrifugal devices were obtained from Sartorius (Palaiseau, France). The Oasis^®^ HLB (Hydrophilic Lipophilic Balance) 96-well extraction plates were obtained from Waters (Milford, MA, U.S.A.). Acetonitrile (HPLC grade) was purchased from Carlo Erba (Val de Reuil, France). The BCA protein assay kit was obtained from Pierce Biotechnology (Rockford, IL, U.S.A.). Quantified standard mixtures of peptides and proteins were purchased from Bruker Daltonics Gmbh (Bremen, Germany). All other reagents and chemicals were purchased from Sigma Diagnostics (St. Louis, MO, U.S.A.).

### 2.2. Animals and biological samples

Fifteen male Sprague Dawley rats (Janvier CERJ, Le Genest-St-lsle, France) were housed individually in a temperature-controlled room (25 ± 1 °C), with constant photoperiod (12/12, light/dark). They were given free access to water and to a standard diet (50% carbohydrate, 5% fat, and 24% protein, in mass percentage), and allowed to acclimatize for several days. The rats were maintained and treated according to the local Ethics Committee and CNRS (Centre National de la Recherche Sci-entifique) guidelines for the care and use of laboratory animals.

When the rats reached 439.9 ± 2.8 g, blood samples were collected into potassium-EDTA vacutainer^™^ tubes and plasma were prepared through a standardized protocol. After withdrawal, whole blood samples were centrifuged for 10 min at 4 °C and at 3000 rpm (swing bucket rotor) to perform a first separation of plasma from blood cells. Plasma was then centrifuged again for 10 min at 10000 rpm (fixed angle rotor) to remove any residual cells (like platelets), particulates and/or lipids. The fifteen plasma samples were then pooled and aliquots were stored at −80 °C.

### 2.3. Plasma sample preparation

**Figure 1:**
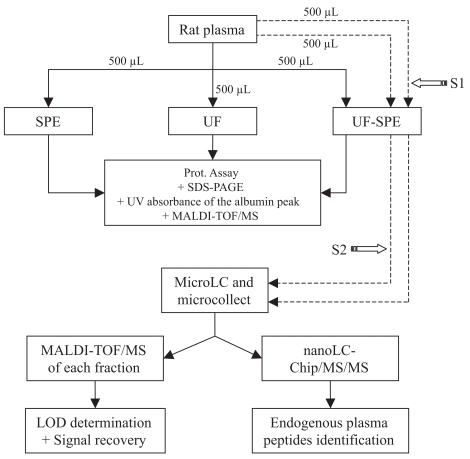
Flowchart of plasma sample preparation and analyses. Three protocols were used to fractionate 500 μL of rat plasma. The SPE protocol was performed using Oasis^®^ HLB 96-well extraction plates; the UF protocol was performed using Vivaspin^®^ 2 30,000 MWCO centrifugal devices; the UF-SPE protocol was a combination of the SPE and UF protocols. Total protein concentration, SDS-PAGE analysis, estimation of albumin depletion by UV absorbance and MALDI-TOF/MS were determined to compare crude plasma samples to SPE eluates, UF filtrates, and UF-SPE eluates. In a separate experiment, we performed LOD determinations and peptide signal recovery in plasma samples using the UF-SPE protocol followed by microLC fractionation by spiking crude plasma (S1) and UF-SPE eluates (S2) with standard peptides. To this end, UF-SPE eluates were further fractionated using a capillary LC system (equipped with a micro fraction collector). Each fraction was then analyzed by MALDI-TOF/MS, and by nanoLC-Chip/MS/MS to identify and characterize endogenous plasma peptides.

Three protocols of plasma sample preparation were compared (Fig. 1). All experiments were conducted in duplicate.

In the first protocol (SPE), 500 μL of pooled plasma samples were diluted in an equal volume of 50 mM aqueous acetic acid and incubated for 15 min at room temperature. Each well was preconditioned with 1 mL of acetonitrile, followed by 1 mL of 50 mM aqueous acetic acid. As the binding capacity of the phase contained in a well is limited (20 μg of protein per mg of phase sorbent), each diluted sample was equally distributed to four wells of an Oasis^®^ HLB extraction plate to limit overcharge. Samples were pulled through the plate using a vacuum pump. The plate was then washed with 1 mL of a 50 mM aqueous acetic acid solution that contained 5% (v/v) acetonitrile. Elution was performed with 200 μl of a 50 mM aqueous acetic acid solution that contained 50% (v/v) acetonitrile. The four eluates of a same sample were then pooled and 0.5 μL of the resulting solutions were spotted on a stainless steel MALDI target plate (Sample Plate 384; Bruker Daltonics Gmbh, Bremen, Germany).

In the second protocol (UF), 500 μL of pooled plasma samples were diluted in 1500 μL of water that contained 10% (v/v) acetonitrile, and then incubated at room temperature for 30 min. Ultrafiltration membranes were conditioned with 1 mL of water and then 2 mL of water that contained 7.5% (v/v) acetonitrile. Samples were transferred to centrifugal devices and centrifuged for 60 min at 6000 rpm (fixed angle rotor) in order to deplete high molecular weight proteins. 0.5 μL of the resulting filtrates were spotted on a stainless steel MALDI target plate.

In the third protocol (UF-SPE), 500 μL of pooled plasma samples were also prepared using Vivaspin^®^ 2 centrifugal devices as described above. The filtrates were further fractionated using Oasis^®^ HLB extraction plates as described above, except that diluted filtrates were applied to only one well of an Oasis^®^ HLB extraction plate due to their lower protein concentration compared to plasma. 0.5 μL of eluates were spotted on a stainless steel MALDI target plate.

For all three protocols, total protein concentration of prefractionated samples was determined using a BCA protein assay kit and 20 μg of proteins were subjected to SDS-PAGE analysis (Fig. 1).

### 2.4. 1-D SDS-PAGE

SDS-PAGE analyses were carried out using 15% SDS polyacrylamide gels in a MiniProtean 3 cell (Biorad, Hercules, CA, U.S.A.). After denaturation by boiling for 5 min, 20 μg of proteins were applied per lane and electrophoresed for 60 min at 140 V. The gels were stained O/N with Phastgel^™^ Blue R (Pharmacia Biotech AB, Uppsala, Sweden). After 3 washings, gel images were acquired with a GS800 calibrated densitometer (Biorad). The albumin bands were determined by comparison to 6.5–205-kDa molecular weight marker.

### 2.5. Albumin depletion capacity

Albumin depletion capacity of the three protocols of plasma sample preparation were estimated and compared (Fig. 1). Aqueous HSA solutions (1, 2, 10, 20, 40, 100, and 200 μg/mL) were prepared in order to construct calibration curves. Samples were prepared as follows: three dilutions (1:500, 1:400, and 1:250) of crude plasma in water; two dilutions (1:100, and 1:200) of SPE eluates in water; speed-vac concentration of UF filtrates (5- and 10-fold) and of UF-SPE eluates (2.5- and 3-fold).

Relative quantitation was performed by UV absorbance measurement using an Agilent 1100 series liquid chromatograph equipped with an UV-vis detector, the whole HPLC system being controlled by the ChemStation Rev A10.02 software (Wilmington, DE, U.S.A.). 50 μl of aqueous HSA solutions and of the samples (diluted plasma, diluted SPE eluates, and concentrated UF filtrates and UF-SPE eluates) were injected onto the LC reverse-phase column (Vydac 300-C8, 25 cm × 2.1 mm, with a 5 μm particle size). The procedure was conducted in duplicate for each concentration. Mobile phase solvents were water (A) and acetonitrile (B), containing 0.1% and 0.08% TFA, respectively. The chromatographic separation was achieved with the following gradient: 2 min. at 10% B; from 10% to 65% B in 33 min.; from 65% to 90% B in 5 min. finally, 5 min. at 90% B. The column was maintained at 40 °C and the fow rate was set at 250 μl.min. UV absorbance detection was set at 214 nm.

Integration of the peak corresponding to albumin was performed using the ChemStation Rev A10.02 software. Linear regression analysis was used to define calibration curves. Regression analysis was performed using the equation *y* = *ax* + *b*, which was fitted to the data, where *y* represents the albumin area, *x* the albumin injected quantity, *a* the slope of the regression line and *b* the *y*-intercept.

### 2.6. MALDI-TOF/MS analyses

Deposition of 0.5 μL of each sample (see 2.3. and Fig. 1) on stainless steel MALDI target plates was followed by addition of 0.5 μl of a saturated solution of α-cyano-4-hydroxycinnamic acid. Each sample was spotted in quadruplicate. MALDI spectra were obtained using an ULTRAFLEX^™^ mass spectrometer (Bruker Daltonics Gmbh, Bremen, Germany). The instrument was used at a maximum accelerating potential of 25 kV in positive mode, and spectra were acquired both in the reflector and linear modes.

In reflector mode, external mass calibration was performed using a commercially prepared standard mixture of peptides (Bruker Daltonics Gmbh, Bremen, Germany). This calibration of MALDI mass spectra was carried out using singly charged monoisotopic peaks of angiotensin II (m/z = 1046.542), angiotensin I (m/z = 1296.685), substance P (m/z = 1347.736), bombesin (m/z = 1619.823), ACTH_1–17_ (m/z = 2093.087) and ACTH_18–39_ (m/z = 2465.199), and somatostatin (m/z = 3147.471). For each MALDI analysis, 400 spectra were acquired (100 laser shots, at a laser power of 40 ± 5%, at four different spot positions) using the FlexControl^™^ acquisition software (Version 2.2, Bruker Daltonics). Monoisotopic peptide masses of signals with a signal-to-noise ratio >3 in a mass range of 700–4000 Da were automatically annotated using the FlexAnalysis software (Version 2.2, Bruker Daltonics).

In linear mode, external mass calibration was performed using a commercially prepared standard mixture of peptides and proteins (Bruker Daltonics Gmbh, Bremen, Germany). This calibration of MALDI mass spectra was carried out using singly charged average peaks of angiotensin II (m/z = 1047.20), angiotensin I (m/z = 1297.51), substance P (m/z = 1348.66), bombesin (m/z = 1620.88), ACTH_1–17_ (m/z = 2094.46), ACTH_18–39_ (m/z = 2466.73), somatostatin 28 (m/z = 3149.61), insulin (m/z = 5734.56), ubiquitin I (m/z = 8565.89), cytochrom C (m/z = 12361.55), and doubly charged average peak of cytochrom C (m/z = 6181.05). For each MALDI analysis, 600 spectra were acquired (200 laser shots, at a laser power of 60 ± 5%, at three different spot positions) using the FlexCon-trol^™^ acquisition software. After baseline subtraction, all signals with a signal-to-noise ratio >20 in a mass range of 1000–15000 Da were automatically annotated using the FlexAnalysis software.

For a given sample, reproducibility of the 4 repeated spots was assessed by calculating the coefficient of variation (CV) of all peak intensities normalized by global intensity of the corresponding spectrum. Subsequently, reproducibility of duplicate experiments was assessed by calculating the CV of averaged intensities of the peaks, calculated for each single experiment. The number of peaks detected in each sample was computed.

### 2.7. MicroLC fractionation

UF-SPE eluates were speed-vac concentrated to 10 μL, and then fractionated using a capillary LC system equipped with a micro fraction collector (Fig. 1), all the system being controlled by the Chem-Station Rev A10.02 software (Agilent 1100 series, Wilmington, DE, U.S.A.). The samples were injected onto a LC reverse-phase column (Zorbax 300SB-C18, 15 cm × 500 μm, with a 5 μm particle size). The procedure was conducted in duplicate for each concentration. Mobile phase solvents were water (A) and acetonitrile (B), containing 0.05% and 0.045% TFA, respectively. The chromatographic separation was achieved with the following gradient: 5 min. at 10% B; from 15% to 60% B in 35 min.; from 60% to 80% B in 5 min. finally, 5 min. at 80% B. The column was maintained at 40 °C and the flow rate was set at 16 μl.min^−1^. Between 2 and 40 min., fractions of 2 min. each were collected in a 96 microwell plate.

Each collected fraction was speed-vac concentrated to 10 μL, and 0.5 μL of the resulting solutions were spotted on a MALDI target and analyzed to follow the peak elution and to specify the fractions containing the spiked peptides. Signal positions of standard peptides (retention time) were determined in control experiments without plasma.

### 2.8. LOD determination

**Figure 2:**
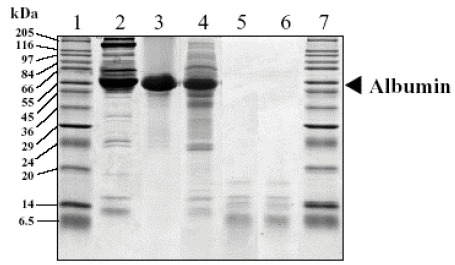
1-D SDS-PAGE analysis of crude plasma (lane 2), commercially prepared HSA (lane 3), SPE eluate (lane 4), UF filtrate (lane 5), and UF-SPE eluate (lane 6). Molecular weight markers (6.5–205-kDa) were electrophoresed in lanes 1 and 7. 20 μg total protein was applied per lane and visualized by coomassie blue staining. The albumin band is shown. See Figure 1 legends for details regarding the SPE, UF and UF-SPE protocols.

It is well known that sample preparation steps generally induce loss of peptides. Knowing that 50 fmol of a given peptide can easily be detected by MALDI-TOF/MS, and estimating arbitrarily and pessimistically that the remaining quantity of a given molecule after the UF-SPE protocol is ten times lower than the initial spiked quantity, we decided to spike plasma samples (500 μL) with 500–10000 fmol (representing approximately 10^−4^–10^−6^% of total proteins, by weight) of a standard mixture of peptides. The main requirements for such a mixture were to know exactly the concentration of composing peptides, which also had to be in a mass range representative of endogenous plasma peptides (1000–3000 m/z). For these reasons, we used the peptide standards provided by Bruker Daltonics (see above). We performed LOD determinations of spiked peptides in plasma samples using the UF-SPE protocol followed by microLC fractionation (Fig. 1). Peptides were spiked 1) directly in crude plasma before its entering in the UF-SPE protocol (S1, see Fig. 1), and 2) in UF-SPE eluates (S2, see Fig. 1). The procedure was conducted in duplicate for each concentration. LOD determination was based on detection of a given peptide on MALDI mass spectra. An estimation of peptide signal recovery was also obtained by comparing peak intensities between S1 and S2.

### 2.9. NanoLC-Chip/MS and MS/MS

NanoLC-Chip/MS and MS/MS of the peptides contained in microLC fractions collected from UF-SPE eluates were performed (Fig. 1) on an Agilent 1100 Series HPLC-Chip/MS system (Agilent Technologies, Palo Alto, U.S.A.) coupled to a HCT^™^ Ultra ion trap (Bruker Daltonics Gmbh, Bremen, Germany).

The chromatographic separations of the microLC collected fractions were conducted on an HPLC chip composed of a 40 nL enrichment column and a reverse-phase separation column (Zorbax 300SB-C18, 15 cm × 75 μm, with a 5 μm particle size). Mobile phase solvents were water (A) containing 2% acetonitrile and 0.1% HCOOH and acetonitrile (B) containing 2% water and 0.1% HCOOH. The chromatographic separation was achieved with the following gradient: from 8% to 40% B in 30 min. from 40% to 70% B in 1 min. finally, 3 min. at 70% B and the flow rate was set at 300 nl.min^−1^.

The mass spectrometer was operated in positive mode by applying a voltage of −1850 V to the capillary cap. The fractions were initially analyzed in full scan mode. A total of 4 scans were averaged to obtain a MS spectrum (scan speed 8100 m/z per sec.). Using an automatic switching between MS and MS/MS modes, MS/MS fragmentation was performed on the three most abundant doubly or triply charged ions on each spectrum, using collision-induced dissociation. A total of 6 scans were averaged to obtain a MS/MS spectrum (scan speed 26000 m/z per sec.). The complete system was fully controlled by the ChemStation Rev B.01.03 (Agilent, Wilmington, DE, U.S.A.) and EsquireControl 6.0 (Bruker Daltonics Gmbh, Bremen, Germany) softwares.

For all MS/MS experiments, generated fragment peak lists were submitted to a local Mascot server (Mascot version 1.9, Matrix Science, London, U.K.). The searches were performed against the NCBInr database, and Mascot search parameters were set as follows: no enzyme was selected, taxonomy was restricted to the Rattus, oxidation of M, N-term acetylation and N-acetylation of proteins were set as variable modifications, the peptide mass error tolerance was restricted to 0.25 Da in both MS and MS/MS modes. Automatic *de novo* sequencing was performed using the PEAKS Studio 3.0 software (Bioinformatics Solutions Inc., Waterloo, Canada) with the same parameters used for Mascot search. All deduced sequence tags were submitted to the NCBI BLAST (http://130.14.29.110/blast/) program to perform sequence similarity searches in the available databases and thus identify the peptide. The identifications were validated according to the established guidelines for proteomic data publication (Carr, 2004; Bradshaw, 2005; Wilkins, 2006).

### 2.10. Statistical analysis

Values are presented as means ± SEM of 2–4 determinations per protocol. Student’s t-test was used to compare the total protein amount and depletion, and estimated albumin amount and depletion (p < 0.05).

## 3. Results and Discussion

### 3.1. Characterization of the three plasma sample preparation protocols

A commercial protein assay kit (BCA) was used to determine the concentrations of total proteins in crude plasma, UF filtrates and SPE and UF-SPE eluates. From these total protein concentrations, the total amount of proteins was calculated in crude plasma and in prepared samples through the three protocols to estimate the loss of total plasma proteins. Hence, the protein amount calculated after UF, SPE and UF-SPE was significantly lower (2.7-fold, p = 0.0003; 21.9-fold, p < 0.0001; and 235.5-fold, p < 0.0001, respectively; Table 1) than in crude plasma. Total protein amount was significantly reduced by UF compared to SPE (8-fold, p = 0.0005), UF-SPE compared to SPE (87-fold, p = 0.0003) and UF-SPE compared to UF (11-fold, p < 0.0001). Total protein depletion from plasma samples was then significantly greater with the UF and UF-SPE (1.5–1.6-fold; p < 0.0001; Table 1) than the SPE protocol, and the combination of UF and SPE depleted significantly more plasma proteins than UF alone (p < 0.0001).

Effectiveness of ultrafiltration to successfully remove high molecular weight proteins is illustrated by 1-D SDS-PAGE where bands with a molecular weight more than 20,000 Da (lanes 5 and 6 of the Coomassie blue stained gel, Fig. 2) can not be observed. The step of membrane filtration is then strongly recommended to efficiently deplete a maximum of high molecular weight proteins from plasma. Plasma is believed to be composed of more than 10,000 different proteins and peptides with a wide range of concentrations that spans at least ten orders of magnitude (from millimolar to sub-picomolar concentrations). The 22 major (“high abundance”) proteins occupy approximately 99% of the total plasma proteome (Anderson and Anderson, 2002), the majority of all circulating proteins and peptides being then expected to be present at very low concentrations (“low abundance”) (Adkins, 2002). Low abundance and/or low molecular weight molecules are generally those that are bioactive in physiological mechanisms. By depleting more than 99% of plasma proteins, our UF and especially UF-SPE protocols give the possibility to potentially detect low molecular weight proteins and peptides. Consistently, in addition to the depletion of high molecular weight proteins, the appearance of low molecular weight and/or low abundance proteins (<20,000 Da) in the albumin depleted samples by the UF and UF-SPE protocols that were not detected in the nondepleted plasma demonstrates the usefulness and efficiency of such depletion protocols (lanes 5 and 6 of the Coomassie blue stained gel, Fig. 2). Nevertheless, the use of an ultrafiltration step is well known to potentially remove peptides that are strongly bound to carrier proteins, such as albumin. Recovery of peptides can notably be improved by denaturating conditions (Tirumalai, 2003; Harper, 2004; Merrell, 2004). In order to minimize peptide losses, we performed the filtration step on diluted plasma samples with aqueous acetonitrile (10%), an organic solvent that can disrupt hydrophobic interactions. Despite these precautions, some peptide losses may remain. In particular, while it was present in crude plasma (lane 2 of the Coomassie blue stained gel, Fig. 2), the absence of the band between 6.5 and 14 kDa in UF filtrates and UF-SPE eluates (lanes 5 and 6 of the Coomassie blue stained gel, Fig. 2) can be noticed. Further studies should aim at reducing such losses in any fractionation protocol. On the whole, protein depletion through the UF and UF-SPE protocols and SDS–PAGE analysis were reproducible between duplicate experiments with the appearance of new bands below 20,000 Da in the albumin depleted samples. Therefore, we believe that these protocols are well-adapted to explore the plasma low molecular weight and/or low abundance fraction.

**Figure 3:**
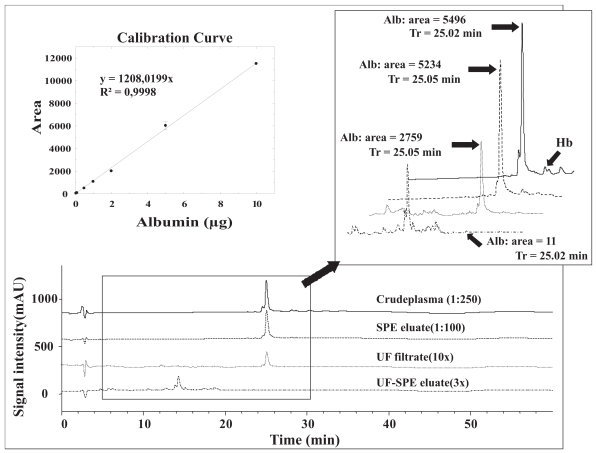
Representative UV-chromatograms of 50 μL of either diluted or concentrated samples. See Figure 1 legends for details regarding the SPE, UF and UF-SPE protocols. UV absorbance detection was set at 214 nm. A calibration curve was constructed using HSA for the determination of albumin (Alb) quantity in crude plasma, SPE eluates, UF filtrates and UF-SPE eluates. Error bars indicate the relative standard error for each sample run in duplicate. Full range and expanded chromatograms of all four samples are shown at the same scale. Integrated areas and retention time of albumin are given. UV peaks corresponding to haemoglobin (Hb) are also shown.

Albumin generally represents about 50% of total plasma proteins (Anderson and Anderson, 2002). Among others, the presence of such a highly concentrated protein is considered as a main obstacle to the detection and characterization of plasma peptides. A host of affinity-based methods have thus been developed to more or less specifically remove albumin from plasma samples. The use of Cibacron Blue resins generally leads to non-specific and modest protein removal (Colantonio, 2005). Albumin depletion kits leads to the depletion of about 70% human plasma albumin (Granger, 2005). Using affinity chromatography, the specific removal of 98%–99% of albumin (Greenough, 2004; Fountoulakis, 2004) and detection of many lower abundance proteins (Pieper, 2003) from human plasma have been reported. However, this approach does not appears applicable to a large number of samples. Various depletion columns have also been tested and were shown to remove 96%–99% of albumin from human serum samples (Björhall, 2005). The Multiple Affinity Removal System (Agilent technologies), composed of beadfixed antibodies, is one of the most successful devices that is designed to remove six to seven interfering major proteins from human plasma samples and three from mouse plasma samples. It reduces the total human serum protein mass by 85% (Martosella), and provides an albumin depletion rate >99% (Björhall, 2005; Brand, 2006). However, such a system does not allow to fractionate simultaneously a high number of samples. Solid phase extraction and membrane filtration are other suitable methods to remove high abundance proteins and better detect the low abundance plasma protein fraction (Tirumalai, 2003; Chernokalskaya, 2004; Souverain, 2004; Koomen, 2005; Orvisky, 2006; Ingvarsson, 2006). To better characterize these latter methods, we evaluated the % albumin depletion that can be obtained by the SPE, UF and UF-SPE protocols that are presented here. To this end, we used various concentrations of aqueous HSA to construct calibration curves, and UV absorbance to detect the albumin corresponding signals (Fig. 3). Hence, we show here that the estimated amount of albumin present in the various samples was lowered in a gradual manner according to the protocol considered (Table 1), being significantly reduced by SPE compared to crude plasma (1.7-fold; p = 0.0198), by UF compared to SPE (728.5-fold; p = 0.0044) and by UF-SPE compared to UF (761.1-fold; p = 0.0106). The use of membrane filtration, alone or in combination with SPE, then provide a significantly more efficient (~2.4 times more; p = 0.0044) albumin depletion (>99.9%) than the use of SPE sorbent alone (42.1%). The combination of UF and SPE depleted significantly more albumin than UF alone (p = 0.0106). In the SPE protocol we decided to distribute the sample to four wells of the Oasis^®^ HLB extraction plate to limit overcharge. The poor albumin depletion capacity of this protocol may be due, in part, to the fact that four wells are not enough. Indeed, about 50 wells should have been used for an unique sample to avoid any overcharge, what we considered incompatible with high throughput analyses. From these results, membrane filtration combined with SPE appear to deplete albumin from plasma samples as efficiently as more specific techniques like the Agilent Multiple Affinity Removal System (see above). Disappearance of the albumin band in the SDS-PAGE analysis confirm the high efficiency of the UF and UF-SPE protocols in depleting albumin (lanes 5 and 6 of the Coomassie blue stained gel, Fig. 2). These protocols have also the advantage to be suitable for high troughput analyses.

### 3.2. MALDI-TOF/MS analyses after the three plasma sample preparation protocols

**Figure 4:**
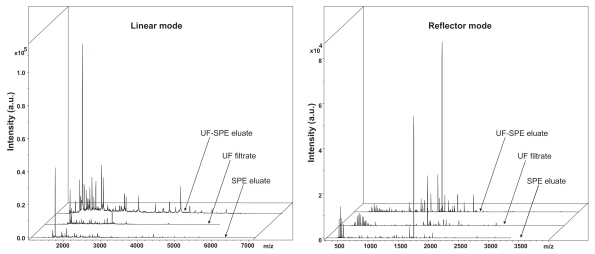
Representative examples of the MALDI-TOF/MS spectra obtained after plasma fractionation by the SPE, UF and UF-SPE protocols. See Figure 1 legends for details regarding these protocols. The molecular weight calculations (*m/z* values) and relative intensities are shown along the *x*- and y-axis, respectively. SPE eluates, UF filtrates, and UF-SPE eluates were analyzed using an Ultraflex instrument (Bruker Daltonics) operated in positive mode. Spectra were acquired both in the linear (left panel) and reflector (right panel) modes.

SPE eluates, UF filtrates and UF-SPE eluates as well as washing solutions (SPE and UF-SPE) were analyzed by MALDI-TOF/MS using an Ultraflex instrument (Bruker Daltonics) operated in positive mode. Spectra were acquired both in the linear and reflector modes. Mass spectra were all dominated by an intense signal at ~1740 m/z (Fig. 4). Peak intensities were much higher after the UF-SPE protocol with more peaks detected, thus indicating a higher sensibility of detection after this protocol. Table 2 summarizes the numbers of peaks that were detected using the three fractionation protocols, in a single experiment or when considering duplicate experiments. Hence, the maximum number of peaks which were detected after the UF-SPE protocol in a single experiment (N_1_ = 77 peaks, Table 2) was higher than after the UF (1.3–1.4-fold) and SPE (1.7–3.8-fold) protocols. In line with these results, about 20–50 peptide peaks were observed for example 1) in a single MALDI-TOF/MS mass spectrum from human serum (1 mL) using ultrafiltration and ZipTip C_18_ desalting as the sample preparation protocol (Chernokalskaya, 2004), and 2) in a SELDI-TOF/MS analysis of human serum (20 μL) after fractionation using anion exchange beads in a 96-well format (Solassol, 2005).

In the current study, with respect to the reproducibility of the 4 repeated spots in each sample in a single experiment, the calculated CVs for all peaks were below 20%–35% in all samples. Such CV values are in line with commonly observed CVs, that are generally calculated for few selected peaks in the literature (Zhang, 2004; Baumann, 2005; Orvisky, 2006). In the present study, CV values were calculated on all peaks, what support strongly the good reproducibility of the three protocols. Consequently, we included all spectra in duplicate experiment comparisons. To perform these comparisons, we set the threshold of acceptable CV between duplicate experiments at 35%. Consequently, 70%–90% of the N_1_ peaks (see Table 2) were considered to be detected in a reproducible manner between duplicate experiments in linear mode and 55%–80% in reflector mode. These percentages, which correspond to the N_2_ peaks mentioned in Table 2, confirm the good reproducibility of the three protocols, the UF and UF-SPE protocols providing the most reproducible data. The majority of the N_2_ peaks (43%–57% in linear mode and 77%–82% in reflector mode) were found in the low mass range (1000–2300 m/z in linear mode and 800–2000 m/z in reflector mode). 32%–36% (linear mode) and 18%–23% (reflector mode) of the N_2_ peaks were found in the medium mass range (2300–3700 m/z in linear mode and 2000–3000 m/z in reflector), the remaining 4% –12% of the N_2_ peaks detected with the linear mode ranging from 3700 to 6000 m/z. Moreover, reproducibility was always better in the low mass range, either in linear or reflector mode. When analyzing also washing solutions from the SPE and UF-SPE protocols, some more peaks below 3600 m/z can be detected (see N_3_ in Table 2) with a CV between duplicates that was, for 60%–100% of them, less than 30%. Enrichment of small peptides were then favoured by all these protocols and their detection has been achieved with great reproducibility.

**Figure 5:**
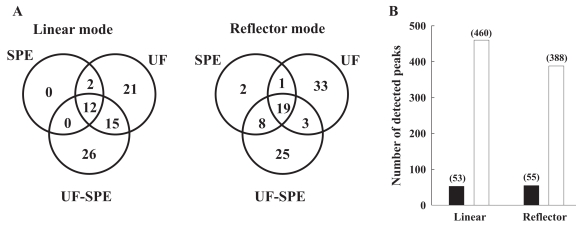
**A.** Comparison of the numbers of peaks detected specifically by a given protocol or by more than a protocol. **B.** Comparison of the number of peaks (mentioned between brackets) detected after UF-SPE followed (open bars) or not (black bars) by microLC fractionation. See Figure 1 legends for details regarding the SPE, UF and UF-SPE protocols. SPE eluates, UF filtrates, and UF-SPE eluates, as well as fractions collected from the microLC system were analyzed by MALDI-TOF/MS using an Ultraflex instrument (Bruker Daltonics) operated in positive mode. Spectra were acquired both in the linear and reflector modes. Peak numbers were deduced from annotated spectra using the FlexAnalysis software (Version 2.2, Bruker Daltonics).

Figure 5 compares the number of peaks that were specifically detected after a given protocol or just as well after more than a protocol. The UF and UF-SPE protocols bring many more informations than the SPE protocol. In addition to the 27 (linear mode) and 22 (reflector mode) peaks that were detected after both the UF and UF-SPE protocols, 23 (linear mode) and 34 (reflector mode) supplementary peaks have been detected specifically after the UF in comparison to the UF-SPE protocol. 26 (linear mode) and 33 (reflector mode) supplementary peaks have also been detected specifically after the UF-SPE in comparison to the UF protocol. As they bring a rather equivalent amount of specific informations, the UF and UF-SPE protocols therefore constitute complementary approaches to detect a maximum of endogenous plasma peptides.

As a consequence of the MALDI ionization procedure which results mostly in singly charged molecules, MALDI-MS spectra are easier to interpret and compare than those from ESI-MS. Therefore, LC MALDI-TOF/MS combination is well-adapted for peptide analysis of complex mixtures such as body fluids (Clynen 2003). Because MALDI-TOF/MS spectra acquired from the UF-SPE protocol provided numerous peak detections with greater peak intensities than the two other protocols, we further fractionated UF-SPE eluates using a microLC system. Following microcollect, a total of 460 (linear mode, up to 6100 m/z) and 388 (reflectror mode, up to 3900 m/z) peptide peaks were then detected in eight different fractions by MALDI-TOF/MS (Fig. 5). About 64% (linear mode) and 94% (reflector mode) of these signals were below 3000 Da. Reproducibility of these detections was estimated by calculation of a CV for approximately half of these signals, that were randomly selected. Between duplicate experiments, mean CV values were 31% and 34% for data acquired in linear and reflector mode, respectively. Hence, by reducing the complexity of samples, microLC fractionation greatly improved the number of signals detected in a reproducible manner, in comparison to UF-SPE alone (4–6 times, Fig. 5). Such results are in accordance with previous data showing that, independently of sample volumes, hundreds of peptides can be detected if sample preparation protocols are made up with chromatographic steps, in combination with various other fractionation techniques (Table 3). Moreover, ultrafiltration is always associated with improved detection of low molecular weight signals; the lower the MWCO, the lower the mass range of peak detection.

### 3.3. LOD and peptide recovery after the UF-SPE protocol

Spiked peptide quantities in plasma and UF-SPE eluates (500–10000 fmol) correspond to concentrations of 1–20 nmol/L and 2.5–50 nmol/L, respectively. After UF-SPE and subsequent microLC fractionation, 19 fractions of 32 μl were obtained per sample. Each fraction was then concentrated to 10 μL, and analyzed by MALDI-TOF/MS. Spiked peptides were detected in eight different fractions. The lowest detected quantities for each peptide ranged from 0.5–5 pmoles. Such quantities correspond to spiked concentrations of 1–10 fmol/μL prior to ultrafiltration (S1 in Table 4). We were then able to detect peptides whose spiked quantity represented, by weight, approximately 10 ^−6^% of total plasma proteins in the initial 500 μL of sample.

In a previous work, determination of LOD has been performed after fractionation of plasma samples by ultrafiltration and reverse phase chromatography (Tammen, 2005a). In this latter study, the lowest detected concentration was for example 50 pM for ACTH_18–39_ and substance P, which correspond to a LOD 20–200 times less than found here. This could be due to the fact that we fractionated a volume of plasma 1.5 times less. Apart from the use of different ultrafiltration devices, we also introduced a complementary step of SPE, which could remove an additional amount of materials in comparison to ultrafiltration alone. Moreover, we only collected 19 fractions by micoLC whereas Tammen et al. collected 96 of them (Tammen, 2005a), thus all the more reducing sample complexity. Although SPE is probably the more limiting step of our protocol for LOD to correspond to peptides in concentrations relevant for biology and medicine, it has the advantages of strongly concentrating samples and of allowing high throughput analyses. This is obviously very important in order to be able thereafter to inject the totality of the fractionated samples on a nanoLC/MS/MS system with the aim of identifying the separated molecules, what is essential for any differential analysis in the field of clinical and functional proteomics. Improved LOD to detect peptides representing less than 10 ^−7^–10 ^−8^% of total plasma proteins could then be obtained in the future by fractionating a higher initial volume of plasma, testing other SPE sorbents and multiplying the number of collected fractions. Nevertheless, the larger the number of fractions, the more the protocol will be incompatible with high throughput analyses.

In the same previous work of Tammen et al. determination of peptide recovery was also performed after fractionation of plasma samples, but not with the same mixture of spiked peptides (Tammen, 2005a). Only one peptide below 3000 Da (substance P) was spiked for recovery determination. In their conditions and as measured by ELISA, it was shown that the use of membrane with a 10 kDa MWCO allows a recovery of about 90% for substance P, and only 40% for osteocalcin, a protein of about 6 kDa. The main objective beyond biological sample fractionation, is to render possible mass spectrometry-based high throughput differential analyses, i.e., by MALDI-TOF/MS profiling. Therefore, we believe that it was important to determine recovery of a given peptide in a fractionated but still complex matrix, by mass spectrometry and not by a more sensitive method like ELISA. In other words, our aim was not to define exactly peptide recoveries, but rather to estimate MALDI-TOF/MS signal recovery of spiked peptides. Although it is known that signal intensity of a given molecule obtained in a MALDI-TOF/MS analysis is not strictly dependent on the molecule concentration, it is commonly observed that the signal intensity increases as the amount of molecule deposited on the MALDI target is increased. Such a characteristic notably provides a basis for profiling approaches in the field of biomarker research (Marshall, 2003; Linke, 2004; Tammen, 2005b; Baumann, 2005; Ebert, 2006; Orvisky, 2006). Indeed, a good linear correlation was found here by plotting MALDI-TOF/MS intensities against the spiked quantities (angiotensin I, r^2^ = 0.980; substance P, r^2^ = 0.995; bombesin, r^2^ = 0.992). We compared spiked peptide peak intensities between S1 (spiking of crude plasma) and S2 (spiking of UF-SPE eluates) in order to estimate the loss of signal attributable to fractionation steps (Fig. 1). Recoveries of signals from the spiked peptides varied between 5% to 100% depending on both the peptide and spiked quantity (Table 4). As we did not used ELISA, but MALDI-TOF/MS data to assess signal recovery, it is not surprising that lesser recoveries were found here in the peptide range than in the work from Tammen et al. (Tammen, 2005a), for example for substance P. This could also be due to the different initial sample volume and ultrafiltration devices, additional SPE step and lower number of LC fractions, as mentioned for LOD determinations (see above). Best recoveries were obtained for the smaller peptides. Such a dependence of recovery on the molecular size of the spiked peptide was also observed by Tammen et al. (Tammen, 2005a), and may be due to the membrane used. The characteristics of the devices used should therefore be well known and taken into account in order to choose the fractionation method most adapted to a type of given sample and to the purpose of the study.

### 3.4. NanoLC-Chip/MS/MS analyses after the UF-SPE protocol

Even if hundreds of peptides can be detected (see above) using various sample preparation techniques, only a few studies are also designed to the characterization of these detected peptides. Moreover, “low molecular weight plasma fraction” is a concept that should be considered cautiously. Numerous peptides are indeed detected but the corresponding identified molecules are rather more or less small proteins (>10–50 kDa) than actual endogenous peptides (Tirumalai, 2003; Harper, 2004; Koomen, 2005; Zheng, 2006). Most of the studies aiming at the exploration of the “low molecular weight serum proteome” include a step of enzyme digestion. This is the case for example in the study of Harper et al. (Harper, 2004), which reports the identification of 262 proteins using ultrafiltration, isoelectric focusing and mass spectrometry of 200 μL of serum, and in that of Tirumalai et al. (Tirumalai, 2003), which reports the identification of 341 unique proteins using ultrafiltration of 10 μL of serum, SCX chromatography and microcapillary LC-MS/MS. In these two latter studies, identified proteins are referred to as belonging to the human “low molecular weight serum proteome” despite that less than 2–3 of these are below 3000 Da and less than 7–15 are below 10000 Da. Some other peptides were found to originate from proteolytic fragments of several large proteins. Finally, only a few studies have allowed specific identification of 1–2 small proteins and/or peptides (Tammen, 2005b; Orvisky, 2006).

Although actual low abundance plasma protein, namely leptin and ghrelin, as well as peptides such as bradykinin have already been detected and identified by mass spectrometry (Rose, 2004), this was achieved through an industrial-scale approach requiring hundreds of ml of plasma and separation by chromatographic techniques into thousands of fractions. In this framework, other studies have identified low abundance plasma molecules, but their workflows were not designed for high throughput and/or large screening. Using for example a differential peptide display technique, insulin and C-peptide levels have been identified by mass spectrometry as modulated by oral glucose challenge after extraction by membrane filtration and separation by liquid chromatography into 96 fractions (Tammen, 2005b). An analytical method has also been developed for the quantitation of insulin in blood samples using SPE and liquid chromatography (Darby, 2001). In the low concentrated peptide range, fast and simple protocols reducing sample complexity from 5–1000 μl of serum or plasma have, to our knowledge, led to point out only fibrinopeptide A, which is an endogenous circulating plasma peptide commonly found in the ng/mL range (van Hulsteijn, 1982; Ravanat, 1996), as a potential biomarker (Marshall, 2003; Chernokalskaya, 2004; Ebert, 2006; Orvisky, 2006). In an effort to analyse the true plasma peptidome, we decided to avoid any digestion step. Such a strategy was recently applied to the human “low molecular weight serum peptidome” analysis using ultrafiltration of 500 μL of serum and a hybrid ion trap-Fourier transform mass spectrometer, and led to the identification (MS/MS sequencing) with high confidence of 61 proteins, with some that are highly abundant and have a molecular weight greater than 50 kDa (Zheng, 2006). In the current study, nanoLC-Chip/MS/MS analyses allowed determination of tens of peptide sequence tags, including the spiked peptides (not shown). In order to show that our UF-SPE protocol was as or even more efficient than previously reported works when aiming at the characterization of low abundance plasma peptides, we only present here high confidence identification of low abundant (representing ~10^−6^% of total plasma proteins, by weight) endogenous fibrinopeptides A and B (Table 5). Fibrinopeptides play a key role in haemostasis (Stegnar, 2004). Such molecules can then bring informations on cardiovascular diseases and metabolic-related disorders. In addition to be well-adapted to peptide enrichment, the UF-SPE protocol is then also valuable for low abundance molecules detection and characterization. This is obviously a major advantage when searching biomarkers of various diseases in clinical proteomic studies and/or aiming at getting further insights in various physiological processes. This protocol is moreover adapted to high throughput analyses, which constitute an obvious requisite for differential peptidomic approaches.

Apart from hormones or cytokines, the low molecular weight plasma fraction also consists of proteolytic fragments of high molecular weight proteins which may reflect endogenous proteolytic activities that play critical roles in biological processes (Boire, 2005; Villanueva, 2006). As we did not digested our samples by trypsin, a significant number of peptides having non-tryptic termini were observed. Indeed, plasma contains numerous proteases with a non-tryptic specificity (Richter, 1999). In that sense, our results are consistent with those of several previous studies, which identified a large number of peptides with non-tryptic termini (Adkins, 2002; Tirumalai, 2003; Zheng, 2006). As already observed in a previous study (Zheng, 2006), we identified several peptides that were derived from the same protein/peptide sequence. This was for example the case for fibrinopeptides A and B, the peptides corresponding to their sequence being progressively cleaved at the N- and/or C-termini (Table 5). Cleavages were observed at various residues. Such a result confirm that endogenous proteases are involved in the generation of plasma low molecular weight proteome and peptidome. However, since certain proteins did not exhibit such proteolytic fragments, it is expected that proteases activities are rather specific, and the resulting peptides may then provide characterization of a given pathophysiological state.

In the work of Zheng et al. (Zheng, 2006), some ions with a molecular weight of 3500–7500 Da were seen in the full MS scan using a LTQ-FTMS, but almost none of these were identified by current commercial software such as SEQUEST. Two possible reasons for this were first that a software like SEQUEST has difficulty interpreting CID spectrum from ions with a charge state of 4 or higher (no guidance for proper probability or Xcorr cut off), and second that larger peptides have a greater potential for being post-translationally modified, which would also interfere with database identifications. The same reasons can be at the origin of lack of identifications of some MS-detected ions in our study, despite good MS/MS quality. To perform MS/MS data interpretation, we set the mass tolerance at 0.25 Da, which may be of insufficient precision. Indeed, improving this mass tolerance to 0.0 Da by using for example an ion trap-Fourier transform mass spectrometer has proven to lead to numerous identifications as obtained in a previous study exploring endogenous human serum peptidome (Zheng, 2006). It could also be that the corresponding protein and/or peptide is absent of protein databases, for example if the peptide sequence tag do not correspond to any proteolytic fragment or if no protein precursor is known. Finally, it is possible that too short sequences may impair identifications using the BLAST programs.

## Conclusion

In this study, we developed simple protocols of plasma sample prefractionation that are adapted for high throughput analyses since quite all steps were automatised and would be capable of processing up to 96 samples in parallel. An efficient separation of low and high abundance molecules was obtained as illustrated by the estimation of a >99% albumin depletion, and by enrichment of more than 450 peptides (<3000 m/z). Details on MADI-TOF/MS spectra show good reproducibility (CV < 30%–35%) of detections through fractionation protocols combining ultrafiltration and SPE. MALDI-TOF/MS-determined LOD, as low as 1 fmol/μL for spiked peptides representing ~10^−6^% of total proteins by weight, and signal recovery of spiked peptides were shown to depend on the peptide and concentration. In addition to being focused on the enrichment of peptides below 3000 Da we also had an effort to get sequence informations. By MS/MS sequencing, we were able to identify spiked peptides and also low abundance peptides, namely fibrinopeptides A and B, as efficiently or even more than other studies. Such peptides can bring important informations regarding cardiovascular diseases and metabolic related disorders. Altered patterns of progressively cleaved peptides from a single protein could also be of valuable importance in the characterization of a given pathophysiological state.

In an attempt to explore, in the future, the low molecular weight plasma fraction, the use of a fractionation protocol like UF-SPE therefore would be of particular interest in the context of differential profiling analyses. To detect and characterize more and more low abundance molecules, such a protocol would also be developed to fractionate higher initial plasma volumes.

## Figures and Tables

**Table 1:** Total protein amount and estimated albumin amount in plasma samples.

	Crude plasma	SPE	UF	UF-SPE
Plasma sample volume (μL)	500	500	500	500
Eluates or filtrates volume (μL)	-	800	2000	200
Total protein concentration in sample (mg/mL)	59.40 ± 1.10	-	-	-
Total protein concentration in eluates or filtrates (μg/mL)	-	13.86 ± 0.45*	0.68 ± 0.04*†	0.64 ± 0.01*†
Total amount of proteins in sample (μg)	29.70 ± 0.32	-	-	-
Total amount of proteins in eluates or filtrates (μg)	-	11.09 ± 0.36*	1.36 ± 0.04*†	0.13 ± 0.01*†◘
% protein depletion	-	62.65 ± 0.39	95.75 ± 0.04†	99.57 ± 0.01†◘
Estimated amount of albumin in sample (mg)	13 ± 1	-	-	-
Estimated amount of albumin in eluates or filtrates (μg)	-	7769 ± 518*	10 ± 1*†	<1*†◘
Estimated % albumin depletion	-	42.1 ± 3.8	99.9 ± 0.1†	>99.9†◘

Crude plasma samples were compared to the eluates and filtrates obtained through three plasma sample preparation protocols. See Figure 1 legends for details regarding the SPE, UF and UF-SPE protocols. Total amount of proteins in crude plasma samples, UF filtrates and SPE and UF-SPE eluates were calculated from total protein concentrations determined by a BCA protein assay kit. Estimated amount of albumin in crude plasma samples, UF filtrates and SPE and UF-SPE eluates were deduced from relative quantitation performed by UV absorbance using an Agilent 1100 series HPLC system (see Fig. 3 for calibration curve). Values are means ± SEM of 2–4 determinations per protocol.

* significantly different from crude plasma (p < 0.02).

† significantly different from SPE (p < 0.02).

◘ significantly different from UF (p < 0.02).

**Table 2:** MALDI-TOF/MS analyses of plasma samples processed by the SPE, UF, and UF-SPE protocols.

	Linear mode			Reflector mode		
	SPE	UF	UF-SPE	SPE	UF	UF-SPE
Number of peaks (N_1_)	20	55	77	54	70	93
CV (%)	<33	<25	<24	<20	<35	<32
Number of peaks (N_2_)	14	50	53	30	56	55
Number of peaks (N_3_)	6	-	14	6	-	24

Three plasma sample preparation protocols were compared. See Figure 1 legends for details regarding the SPE, UF and UF-SPE protocols. MALDI-TOF/MS analyses were performed in positive mode using an Ultraflex instrument (Bruker Daltonics). Spectra were acquired both in the reflector and linear modes. N_1_ is the maximal number of peaks detected in a single experiment in SPE eluates, UF filtrates and UF-SPE eluates. N_2_ is the number of peaks detected in SPE eluates, UF filtrates and UF-SPE eluates with a CV less than 35% between duplicate experiments. N_3_ is the number of supplementary peaks that could be analyzed if considering also washing solutions. CV corresponds to calculated coefficient of variations of all peak intensities normalized by global intensity of the corresponding spectrum in a single experiment. Peak numbers were deduced from annotated spectra using the FlexAnalysis software (Version 2.2, Bruker Daltonics) with a signal-to noise ratio >20 and >3 in linear and reflector mode, respectively.

**Table 3:** Summary of serum and/or plasma peptide detections and identifications in the literature.

		Fractionation protocol				
Reference	Plasma (μL)	UF (MWCO)	SPE	Chromatography	Other	N° of peaks detected
Baumann, 2005	5	-	no	C3, C8 and C18 magnetic beads	-	Hundreds (<10 kDa)
Orvisky, 2006	15	10–50 kDa	no	C8 magnetic beads	SELDI	Hundreds (<5 kDa)
Koomen, 2005	20	-	yes	RP-C 18 LC	-	Hundreds (<5.5 kDa)
Zheng, 2006	500	10 kDa	no	RP-C 18 LC	ZipTip C18 desalting	Hundreds (<3.5 kDa)
Merrell, 2004	1000	30 kDa	no	RP-POROS R1 Capillary LC	Acetonitrile precipitation	Hundreds (<2 kDa)
Tammen, 2005a, 2005b	1300	50 kDa	no	RP LC	-	Hundreds (<15 kDa)

In these six studies (see complete references in the text), peak detections were achieved through MALDI-TOF/MS and/or SELDI-TOF/MS analyses. UF, ultrafiltration.

**Table 4:** Spiked peptides with their monoisotopic masses, amino acid sequences, retention time, limit of detection and peptide signal recovery.

Name	[M+H]^+^	Sequence	Tr. (min.)	LOD (fmol/μL)		Recovery (%)
				S1	S2	
Angiotensin II	1046.54	DRVYIHPF	18.3	10	5	89–100
Angiotensin I	1296.68	DRVYIHPFHL	21.8	1	2.5	11–50
Substance P	1347.74	RPKPQQFFGLM-NH2	22.9	1	2.5	5–50
Bombesin	1619.82	p-EQRLGNQWAVGHLM-NH2	21.6	10	5	9–12
ACTH_18–39_	2465.20	RPVKVYPNGAEDESAEAFPLEF	24.0	10	25	8–33
Somatostatin 28	3147.47	SANSNPAMAPRERKAGCKNFFWKTFTSC	23.2	-	25	-

Various quantities of peptides from a commercial mixture were spiked 1) directly in the crude plasma before its entering in the UF-SPE protocol (S1), and 2) in the UF-SPE eluates (S2). Before MALDI-TOF/MS analysis, a microLC fractionation was performed. See Figure 1 legends for details regarding the UF-SPE protocol. MALDI-TOF/MS analyses were performed in positive mode using an Ultraflex instrument (Bruker Daltonics). Limit of detection (LOD) and peptide signal recovery for each spiked concentrations were determined from TIC-normalized intensities, using the FlexAnalysis software (Version 2.2, Bruker Daltonics). Retention times (Tr.) of standard peptides were determined in control experiments without plasma.

**Table 5:** Identification of rat plasma fibrinopeptides A and B using a nanoChip-LC/MS/MS instrument and the NCBInr database.

ID	AC	Peptide mass [M]	Observed ion [M+nH]^n+^	Charge	Ion score	Peptide sequence
Fibrinopeptide A	650771P	1105,56	553,79	2+	38	EF**IEAGGD**IR
		1192,59	597,30	2+	62	SE**FIEAGGD**IR
		1293,64	647,83	2+	73	TS**EFIEAGGD**IR
		1394,69	698,35	2+	96	TT**SEFIEAGGD**IR
		1451,72	726,87	2+	81	GTTS**EFIEAGGD**IR
		1552,76	777,39	2+	103	TGT**TSEFIEAGGD**IR
		1667,83	834,92	2+	108	DTGT**TSEFIEAGGD**IR
		1738,86	870,44	2+	144	ADT**GTTSEFIEAGGD**IR
		1738,88	580,63	3+	23	ADTG**TTSEF**IEAGGDIR
		1582,59	792,30	2+	56	ADTGT**TSEFIEA**GGDI
Fibrinopeptide B	650771AE	1061,54	531,78	2+	20	DSD**KVDL**SIA
		1079,54	540,78	2+	37	**TTDSDKVD**LS
		1150,57	576,29	2+	40	ATTDSD**KVDL**S
		1162,58	582,30	2+	46	TD**SDKVDL**SIA
		1318,66	440,56	3+	35	TDSD**KVDL**SIAR
		1318,66	660,34	2+	46	TDSD**KVDL**SIAR
		1334,66	668,34	2+	23	ATTDSD**KVDL**SIA
		1419,70	474,24	3+	50	TTDSDK**VDL**SIAR
		1419,70	710,86	2+	101	TT**DSDKVDL**SIAR
		1490,74	746,38	2+	105	AT**TDSDKVDL**SIAR

Peptide sequences were observed in MASCOT results (ion scores are given in the table), and confirmed by *de novo* sequencing (in bold) using the PEAKS Studio 3.0 software (Bioinformatics Solutions Inc.). Blast E values were commonly ≤1 (≤0.2 for 75% of the searches).

## Abbreviations

SPE: solid phase extraction
UF: ultrafiltration
UF-SPE: combination of ultrafiltration and solid phase extraction
HSA: human serum albumin
CV: coefficient of variation
LOD: limit of detection
Tr: retention time
